# Live Intraoral *Dirofilaria repens* of Lower Lip Mimicking Mucocele—First Reported Case from Croatia

**DOI:** 10.3390/ijerph19074330

**Published:** 2022-04-04

**Authors:** Ivana Skrinjar, Vlaho Brailo, Bozana Loncar Brzak, Jelena Lozic Erent, Suzana Bukovski, Danica Vidovic Juras

**Affiliations:** 1 Department of Oral Medicine, School of Dental Medicine, University of Zagreb, 10000 Zagreb, Croatia; skrinjar.ivana@gmail.com (I.S.); loncar@sfzg.hr (B.L.B.); dvjuras@outlook.com (D.V.J.); 2 Department of Oral Medicine, University Clinical Hospital Zagreb, 10000 Zagreb, Croatia; 3 Department of Clinical Microbiology, University Hospital for Infectious Diseases, 10000 Zagreb, Croatia; jlozic@bfm.hr (J.L.E.); sbukovski@bfm.hr (S.B.); 4 Faculty of Dental Medicine and Health Osijek, Josip Juraj Strossmayer University of Osijek, 31000 Osijek, Croatia; 5 School of Medicine, Catholic University of Croatia, 10000 Zagreb, Croatia

**Keywords:** dirofilariasis, oral, parasitic infestation, mucocele, Croatia

## Abstract

Dirofilariasis is an endemic infestation in tropical and subtropical countries caused by about 40 different species. It rarely occurs in the oral cavity and is mostly presented as mucosal and submucosal nodules. Differential diagnoses include lipoma, mucocele, and pleomorphic adenoma. We report a rare case of oral dirofilariasis mimicking mucocele in a 41-year-old male patient from Croatia without an epidemiological history of travelling outside the country. He came in because of non-painful lower lip swelling that had lasted for two months. The parasite was surgically removed from the lesion. This is the first reported case of oral dirofilariasis in Croatia. It is important to point out this rare diagnosis in order to make dentists aware of the possibility of the presence of such an infestation in common lesions of the oral mucosa.

## 1. Introduction

Dirofilariasis is an endemic zoonotic infestation in tropical and subtropical countries caused by about 40 different Dirofilaria species [1]. Humans are dead-end hosts and are infected via mosquitos most commonly by *Dirofilaria repens* and Dirofilaria immitis [2]. Infestations with *Dirofilaria repens* are more common in Africa, Asia, and Europe in contrast to the Americas, where Dirofilaria immitis predominate [3]. Infestation with Dirofilaria immitis can affect the lungs, causing coughing, chest pain, fever, and pleural effusion. Outside of the lungs, it can be found in the brain, eyes, and testicles. Opposite to Dirofilaria immitis, *Dirofilaria repens* can be found under the skin and mucous membranes and does not affect internal organs [4]. There is evidence that *D. repens* has spread faster than D. immitis from the endemic areas of southern Europe to northern Europe. Climate change, affecting mosquito vectors, and the facilitation of pet travel seem to have contributed to this expansion [5]. The most important reservoir of infection are microfilaremic dogs [6]. In these hosts, the adult worms are usually in the subcutaneous tissue, while microfilariae circulate in the blood. Sexual maturity and female production of microfilaria occur in the vertebrate host, usually in subcutaneous tissue or in muscle sheaths. Several species of mosquito vectors then ingest microfilariae during their bloodfeeding. Following ingestion, microfilariae undergo transformation into third-stage larvae within the intermediate host. The larvae then migrate from the abdomen through the thorax and eventually to the salivary glands of the vector. The transmission of infection to a human host may occur at a subsequent blood meal [7].

Endemic *Dirofilaria repens* infections have been found in dogs in most European countries and, according to Capelli et al. [5], Croatia is listed as an endemic part. The highest incidence in humans has been recorded in Mediterranean countries (Greece, Southern France, and Italy), of which Croatia is also a part.

Diagnosis is usually made by microscopic and macroscopic examination of the worm or by histopathological analysis, which shows a thick, multi-layered cuticle with longitudinal ridges [8].

In a search of the literature on Pubmed, we found only 24 reported cases of Dirofilaria repens involving the oral cavity, with five cases from European countries (Table 1). 

Most of the cases were mucosal or submucosal nodules, and one case was similar to ours [17]. This was a case regarding a 53-year-old male patient from Sri Lanka. The lesion was firm in consistency and normal in colour. The findings were similar to those in our patient.

Pupic-Bakrac et al. [25] performed an analysis since the first case in 1996 and found 30 cases of human *Dirofilaria repens* in Croatia. A total of 16 cases were from the continental regions and 14 were from the coastal regions. Regarding anatomical location, 13 cases were subcutaneous, 12 were ocular, and 5 were found in reproductive organs. None of the cases involved the oral cavity.

An increasing number of cases reported in the literature point toward the trend of human dirofilariasis becoming an emerging zoonosis. However, dental clinicians are usually unaware of the existence of such an infestation [12]. The oral cavity is rarely involved and is mostly presented as mucosal and submucosal nodules [10], and differential diagnoses include lipoma, mucocele, and pleomorphic adenoma. Thus, it is important to point out this rare diagnosis in order to make dentists aware of the possibility of the presence of such an infestation in common lesions of the oral mucosa.

According to the Pubmed search, this is the first case of oral *Dirofilaria repens* reported in Croatia, Europe.

## 2. Case Report

We report a rare case of human oral *Dirofilaria repens* infestation. A 41-year-old male came to the Department of Oral Medicine, School of Dental Medicine University of Zagreb, Croatia in May 2021 due to non-painful lower lip swelling that had lasted for two months. According to the patient′s subjective perception, the swelling occasionally increased and decreased during that time without any known cause. Two weeks before the patient came to our department, the lesion did not change in size or colour. The patient′s medical history showed hypertension, and he was taking an angiotensin-converting enzyme inhibitor.

**Figure 1:**
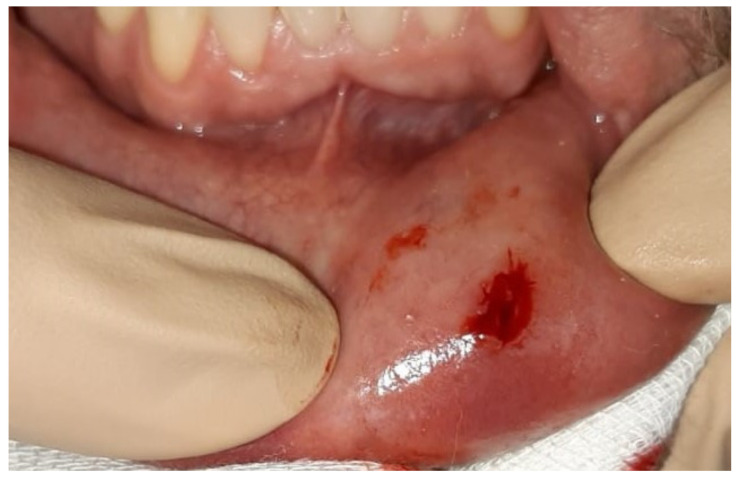
Wound on lower lip after surgical extraction of the parasite.

**Figure 2:**
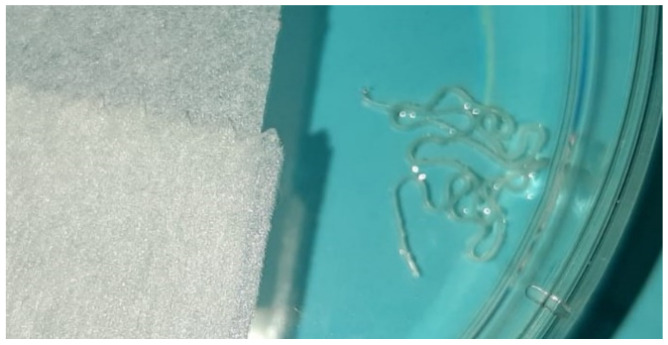
Parasite in saline in the Petri dish.

A clinical examination revealed mild spherical swelling of the lower lip approximately 5 mm in diameter, and a clinical diagnosis of mucocele was established. The change was slightly hard on palpation, indicating an excisional biopsy was required. The patient received local anaesthesia and surgery started. During the procedure, a long thin formation was extracted, and we assumed it was a parasite. The parasite was structureless, measuring 12 cm in length and 0.5 mm in diameter, with a greyish-white colour. The wound of the lower lip after surgery is shown in Figure 1; two sutures were placed at the site. We put the parasite into saline in a Petri dish (Figure 2) and delivered it to the University Hospital for Infectious Diseases, Zagreb, Croatia, immediately. Macroscopic and microscopic analysis showed that it was *Dirofilaria repens*.

The patient’s epidemiological data did not show travel outside of Croatia. The patient stated a trip to the peninsula of Peljesac in the southern part of Croatia ten months before. The patient lives in a household where there are two dogs and a cat.

We also took a complete blood count of the patient, which was found to be normal. After our surgical treatment, the patient was examined by a specialist in infectology and parasitology. According to the diagnosis of *Dirofilaria repens*, which does not affect internal organs, no further treatment was indicated. The patient came to remove the sutures one week after and normal healing was detected. The patient came to check-up appointments after two and six months, and he was healthy with no signs of recurrence.

The patient gave his full written consent form for the publication of his data and the consent is attached to the submission.

## 3. Discussion

This is the first case reporting oral *Dirofilaria repens* infestation in Croatia. Since the first described case of *Dirofilaria repens* in Croatia in 1996, 30 cases of infestation have been recorded [25]. None of these cases affected the oral cavity. Regarding the patients’ contact with animals, in 19 cases, contact was unknown, 3 cases had contact with dogs and cats, two cases had contact with dogs, one case had contact with the domestic animals, one with a dog and a domestic animals, one case had contact with a cat and three cases had no contact with animals. Regarding the patients’ travel history, 17 cases were unknown, 2 had travelled internationally (France and Kosovo), 6 travelled domestically, and 6 patients reported no travel. Our patient reported travelling to the southern part of Croatia (peninsula of Peljesac) and had house contact with a dog and a cat.

The majority of human cases of *Dirofilaria repens* in Europe have been described in the Mediterranean parts of Italy, France, and Greece, and in some Eastern European countries, such as Ukraine, the Russian Federation, and Belarus [5,26]. Searching the Pubmed site with the keywords “*oral dirofilariasis repens*”, we found 24 cases reporting infestations of the oral cavity worldwide. Our search showed six cases of human oral *Dirofilaria repens* from the European part of the world. Two of them were from France [9,23], and one each was from Italy [13], Russia [18], Serbia [22], and Bulgaria [24]. Most of the reported cases were from Sri Lanka (nine cases) [11,17] and the rest were from India (five cases) [1,10,12,15,16], Brazil (two cases) [19,20], the United States (one case) [21], and China (one case) [14], confirming tropical parts of Asia as endemic areas of *Dirofilaria repens* infestation. Most of these cases presented as non-painful swelling of the buccal mucosa and were suspected benign tumours, such as lipoma, adenoma, or mucocele [17], as in our case. One case presented as painful swelling of the right cheek and a clinical diagnosis of pericoronitis of the lower right wisdom tooth was suspected [10]. In some of the cases, the use of preoperative and postoperative non-invasive ultrasonography as a radiation-free diagnostic procedure was considered. Reda et al. [27] conducted a literature overview, which showed that ultrasound could be used for soft oral tissue diagnosis.

Dirofilariasis can affect people of any age. In their review, Chaudry et al. [10] showed that the mean age of the patients was 39.22 years, with a range of 10 months to 80 years. These findings are also confirmed in the oral *Dirofilaria repens* cases in Table 1, showing the range of affected patients from 4 years to 80 years, with a median of 42 years.

The limitations of case reports, in general, include potential publication bias. Albrecht et al. [28] found that only 5% of case reports and 10% of case series reported treatment failure. Another authors [29] suggested that before deciding on writing a case report, the clinician has to determine if the case report is an appropriate type of article. According to this finding, our case does not have potential bias and could improve the knowledge of dental practitioners.

## 4. Conclusions

We present this case to warn of the possibility of parasitic infestation in cases of recurring swelling of the oral mucosa. In our case, surgical removal of the worm was enough to achieve healing. It is important that clinicians outside endemic countries should also be aware of this rare diagnosis.

## Figures and Tables

**Table 1:** *Dirofilaria repens* infestations involving oral cavity.

Reference	Age Sex	Country of Origin	Clinical Findings
Hennocq et al., 2020 [9]	46 F	South eastern France	Submucosal nodule in the cheek after eyelid creeping dermatitis
Chaudry et al., 2019 [10]	26 M	India	Swelling and pain of the right cheek, suspected pericoronitis of the lower right wisdom tooth
Jayasinghe et al., 2015 [11]	21 M	Sri Lanka	Painless swelling of the left cheek
	57 F	Sri Lanka	Well-circumscribed, partially moveable, firm swelling of the left side of the buccal mucosa
Spadigam et al., 2018 [12]	37 F	India	Right buccal vestibule, 1 cm in diameter, swelling
Kurup et al., 2013 [1]	54 F	India	Swelling of the buccal mucosa without any inflammatory signs
Trunfio et al., 2018 [13]	51 F	Italy	Noninflammatory submucosal nodule in the left maxillary vestibule
To et al., 2003 [14]	42 F	New immigrant to Hong Kong from China	Intraoral swelling located in the right maxillary vestibule adjacent to the premolar apical area
Desai et al. 2015 [15]	32 M	India	Swelling of the buccal mucosa
Balaji S.M. 2014 [16]	19 F	India	Solitary, mobile, diffuse, soft, non-tender nodule, 2 cm lateral and superior to left angle of the mouth
Tilakarante and Pitakotuwage. 2003 [17]	26 F	Sri Lanka	Smooth surface of buccal mucosa, suspected salivary tumour
	80 F	Sri Lanka	Swelling/nodule of buccal mucosa, suspected pleomorphic adenoma/lipoma
	52 F	Sri Lanka	Lump hard in consistency on buccal mucosa, suspected lipoma/calcified lymph node
	28 F	Sri Lanka	Extra osseous firm lump on buccal sulcus suspected minor salivary gland tumour
	04 F	Sri Lanka	Submucosal nodule on buccal mucosa, suspected tuberculosis
	40 F	Sri Lanka	Submucosal nodule on buccal mucosa
	53 M	Sri Lanka	Submucosal change on lower lip, suspected mucocele
Kramer L.H. et al., 2007 [18]	23 F	Russia	Oral cavity nodule
Pereira et al., 2015 [19]	65 F	Brazil	Submucosal nodule of the right buccal mucosa
Daroit N.B. et al., 2016 [20]	65 F	Brazil	Submucosal nodule in buccal mucosa
Vélez-Pérez A et al., 2016 [21]	79 M	USA	Infiltrative mass in the right buccal space
Momčilović S. et al., 2019 [22]	45 M	Serbia	Nodule along the lateral edge of the right maxilla
Tourte-Schaefer and Dupouy-Camet 2020 [23]	42 M	France	Non-tender firm nodule of the right cheek
Velev et al., 2018 [24]	37 M	Bulgaria	Swelling of the left buccal mucosa

## Data Availability

Not applicable.
